# Hydration Mechanism and Microstructure Evolution of Seawater-Based Low-Alkalinity Activated Phosphogypsum Cement

**DOI:** 10.3390/ma19030617

**Published:** 2026-02-05

**Authors:** Weisen Liu, Yanlin Zhen, Yuan Feng, Zhongyu Lu, Jianhe Xie

**Affiliations:** 1 School of Civil and Transportation Engineering, Guangdong University of Technology, Guangzhou 510006, China; 1112209005@mail2.gdut.edu.cn (W.L.); zhenyanlin1@mails.gdut.edu.cn (Y.Z.); fengyuan@gdut.edu.cn (Y.F.); luzy@gdut.edu.cn (Z.L.); 2 School of Intelligent Engineering, Guangdong AIB Polytechnic, Guangzhou 510507, China

**Keywords:** phosphogypsum, low alkalinity, seawater, hydration, rheology

## Abstract

This article proposes a novel preparation method for seawater-based low-alkalinity activated phosphogypsum (PG) cement, aimed at enhancing the performance of multi-waste binder systems using the highly ionic environment of seawater while addressing the cost and alkalinity issues associated with traditional high-alkalinity activators. The effects of partial replacement of ground granulated blast furnace slag (GGBS) with PG (0–15%) and fly ash (FA, 20–50%) on the setting time, rheological properties, microstructure, and compressive strength of seawater-based slurries were investigated. Compared to the control group (pure slag), the samples with a synergistic ratio of 5% PG and 35% FA had a mean compressive strength exceeding 60 MPa at 28 days, comparable to that of the control group, with a significant improvement in flowability. The results demonstrate that the proposed preparation method alters the hydration kinetics of alkali-activated GGBS cement and significantly improves the early and later compressive strength of hydrated samples. In the early hydration phase, seawater ions effectively promoted the rapid nucleation and growth of ettringite (AFt) crystals. The low-alkalinity composite activator induced the formation of a substantial amount of C-(A)-S-H gel. In the later stages of hydration, needle-like AFt crystals intertwined with the gel matrix, further densifying the microstructure. The enhancement of the polymer’s performance is primarily attributable to the key “synergistic enhancement effect” between seawater ions and the low-alkalinity environment. This interaction optimizes the formation pathways of key hydration products and refines the pore structure, providing a solid theoretical foundation for the low-carbon, high-efficiency utilization of PG in marine engineering materials.

## 1. Introduction

The production of traditional ordinary Portland cement (OPC) generates a large amount of carbon dioxide emissions, accounting for approximately 8% of global greenhouse gas emissions [1,2]. Alkali-activated binders have emerged to address this issue. These materials are inorganic polymers formed by the reaction of aluminosilicate precursors (such as industrial waste like slag [3] and fly ash, FA [4]) with alkaline activators. Alkali-activated binders can reduce the carbon footprint of Portland cement production by 60–90% [5,6,7], and because of their excellent mechanical properties and durability, they have become a widely favored green binder material [8,9].

Among the many potential precursor materials, phosphogypsum (PG), an industrial byproduct of wet-process phosphoric acid production, has a massive global stockpile. It not only occupies land but is mainly stored in open-air piles, posing a severe environmental threat to soil and water resources [10,11]. Using PG rich in CaSO_4_ is a more sustainable alternative [12,13]. At 278.15–308.15 K, the solubility of CaSO_4_ in NaCl and MgCl_2_ solutions is two orders of magnitude higher than in freshwater, which is related to the dissolution equilibrium of CaSO_4_ in electrolyte solutions [14], as shown in Equations (1) and (2). Thus, seawater, rich in Na^+^ and Mg^2+^ ions, can effectively promote the dissolution of PG at the same time, freshwater consumption in the concrete industry is staggering, and in freshwater consumption in the concrete industry is staggering, and in freshwater-scarce island or coastal areas, replacing freshwater with seawater for mixing has significant strategic importance [15].

(1) $${{\mathrm{NaSO}}_{4}}^{-}(\mathrm{aq})⇌{\mathrm{Na}}^{+}(\mathrm{aq})+{{\mathrm{SO}}_{4}}^{2-}(\mathrm{aq})$$

(2) $${\mathrm{MgSO}}_{4}(\mathrm{aq})⇌{\mathrm{Mg}}^{2+}(\mathrm{aq})+{{\mathrm{SO}}_{4}}^{2-}(\mathrm{aq})$$

In traditional OPC concrete, the addition of seawater has both positive and negative effects. On the positive side, the high concentration of chloride ions in seawater can act as an accelerator, effectively shortening the setting time of concrete and increasing its early compressive strength [16]. Furthermore, using seawater instead of freshwater can alleviate the pressure on precious water resources in coastal and island construction projects [15]. On the negative side, the high salt content in seawater can lead to problems such as crystal precipitation and unstable volume changes in the OPC system. Unlike traditional cement, alkali-activated materials offer a promising alternative. They can effectively immobilize chloride ions within their geopolymer structure, mitigating the negative effects of seawater and using its ionic components to enhance performance. The various ions in seawater profoundly influence the hydration process of the cementing material system [17]. For example, Cl^−^ in seawater may participate in the hydration process as a reactant, promoting the formation of Friedel’s salt and other layered double Cl-hydroxides [18], thereby altering the pore structure [19]. Mg^2+^ and SO_4_^2−^ may consume alkaline components through precipitation reactions or affect the dissolution and polycondensation efficiency of the precursors by altering the ionic strength [20]. The ions introduced by seawater and the substances leached from PG may produce complex coupling effects, particularly when seawater interacts with the complex components of phosphogypsum (PG), and the competitive or synergistic mechanisms in this multi-ion coexisting environment are not yet fully understood.

Despite some progress in the research on PG-based cements, several challenges remain that hinder their large-scale application. First, the soluble acidic impurities in PG, such as phosphorus and fluoride residues, can significantly delay the hydration reactions of the cementitious material, leading to slow early strength development [21] and in severe cases, even result in failure to set. Second, because of the low reactivity of PG, high-concentration strong alkaline activators (such as high-modulus water glass or concentrated NaOH) have often been employed in previous studies to mitigate the negative effects of PG incorporation [22]. These high-alkaline materials not only increase costs and implicit carbon emissions but also tend to cause alkali–silica reaction (ASR) issues on the surface of the hardened body, compromising the volume stability of the material [23]. More critically, the mechanism of seawater’s role in complex multi-waste geopolymer systems is not yet fully understood. Previous studies have mostly focused on the use of seawater in ordinary Portland cement or single precursor (such as pure ground granulated blast furnace slag, GGBS) geopolymers [4]; few have examined the complex, multi-component, low-alkalinity seawater–PG system. How high concentrations of Na^+^, Cl^−^, and SO_4_^2−^ ions in seawater interfere with or synergize with the migration and transformation of impurity ions in PG in a low-alkalinity environment and whether they alter the formation pathways and stability of major hydration products (such as C-(A)-S-H gel) are still unanswered questions. These key physicochemical mechanisms currently lack systematic theoretical explanations.

To address these issues, this article proposes a low-alkalinity, high-performance seawater-based PG cement preparation method. This method uses GGBS, FA, and unaltered PG to construct a multi-waste binder system, optimizing the nucleation pathways of reactions through the synergistic effects of different active precursors [24]. Unlike traditional high-alkaline activation, a low-alkalinity solid activator composed of sodium carbonate (Na_2_CO_3_) and sodium sulfate (Na_2_SO_4_), with partial replacement of the anhydrous sodium silicate (Na_2_SiO_3_), is used in this method. The aim is to reduce the alkalinity of the system while taking advantage of a highly ionic seawater environment to enhance the reactivity of the multi-waste binder system. This approach enables the efficient use of low-value solid wastes such as PG and FA to achieve excellent mechanical properties without requiring complex pretreatment of the precursors.

## 2. Materials and Methodologies

### 2.1. Raw Materials and Preparation

**Figure 1:**
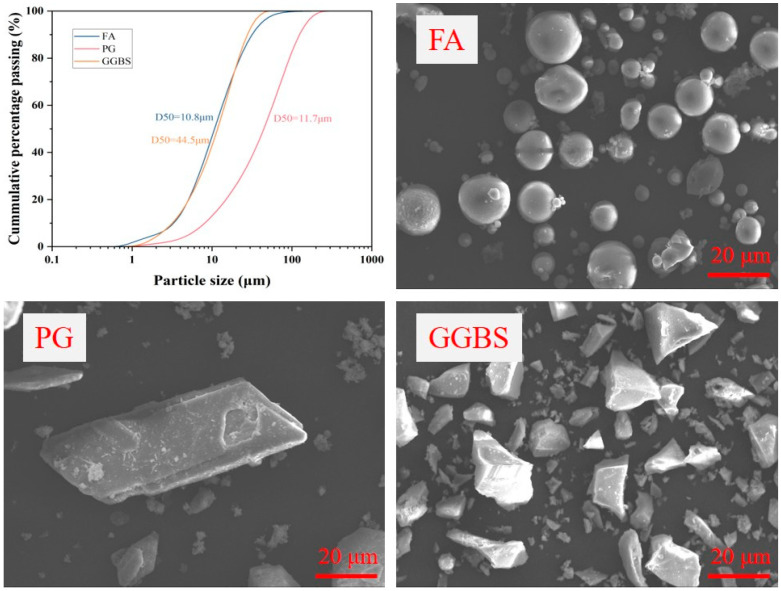
SEM images and particle size distributions of raw materials.

**Figure 2:**
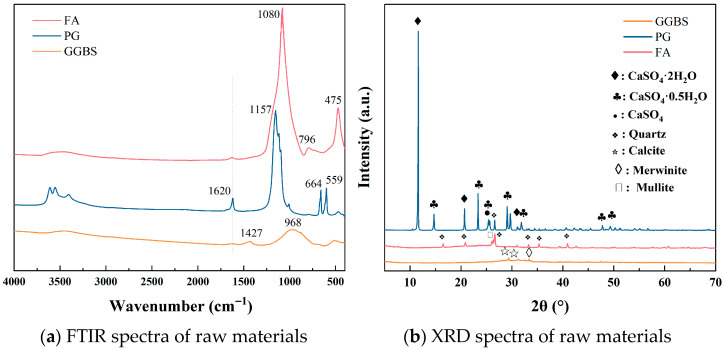
FTIR and XRD spectra of raw materials.

The three solid waste materials used as precursor materials in this study were GGBS, FA, and PG. The unaltered PG was sourced from Hubei Province, China, and the FA and GGBS were purchased from the Gongyi Hengnuo Filter Material Co., Ltd. (Gongyi, China). The PG was dried at 50 °C to a constant weight, crushed to a powder, and soaked in deionized water, resulting in a pH of 2.4. Elemental analysis was conducted using the Mastersizer 3000 X-ray fluorescence spectrometer (XRF) produced by Malvern Company in the Malvern, UK, covering elements from F to U. The results of XRF are shown in Table 1. The main elements in the GGBS were Ca, Si, and Al. The FA was primarily composed of Si and Al. The main components of the PG were Ca and S. Notably, the PG contained a small amount of P_2_O_5_. The microscopic morphology and particle size distributions of the three raw materials are shown in Figure 1. Scanning electron microscopy (SEM) images revealed that the GGBS particles were irregular and angular, the FA particles were smooth and spherical, and the PG were predominantly plate-like in shape. The FTIR and XRD spectra of the raw materials in Figure 2 show that the PG was mainly composed of dihydrate gypsum.

A composite alkaline activator with a Na_2_O content of 4.0 wt%, composed of sodium silicate (Na_2_SiO_3_), sodium carbonate (Na_2_CO_3_), and sodium sulfate (Na_2_SO_4_), was used in this study. The anhydrous Na_2_SiO_3_ powder (Modulus, Ms = 1) was provided by Henan Borun Casting Materials Co., Ltd. (Zhengzhou, China). Both the Na_2_CO_3_ and Na_2_SO_4_ were analytical grade (≥99%) and purchased from Shanghai Aladdin Bio-technology Co., Ltd. (Shanghai, China). To reduce the overall alkalinity of the activator, we selected the inherently mild, near-neutral Na_2_SO_4_ and weakly alkaline Na_2_CO_3_ to replace the high-alkalinity sodium metasilicate. As shown in Table 2, in a seawater environment, the pH of Na_2_SO_4_ and Na_2_CO_3_ solutions are 7.85 and 10.94, respectively, both of which are below 11.0. In contrast, the pH of the Na_2_SiO_3_ solution exceeds 13.5. This reduction in pH is beneficial for the preparation of low-alkalinity phosphogypsum cement, which can mitigate the environmental impact associated with high-alkalinity systems.

The mixing ratios for the Na_2_SiO_3_, Na_2_CO_3_, and Na_2_SO_4_ are shown in Table 3. To prepare the samples, GGBS, FA, PG, and the composite activator powders were first added to a mixing container according to the proportions shown in Table 3 and dry-mixed for 120 s. The corresponding artificial seawater (configured according to Table 4) was then added to the mixing container. The mixture was stirred at low speed for 120 s, followed by high-speed stirring for another 120 s, resulting in a fresh paste. The paste was then poured into 40 mm cubic molds, vibrated for 60 s, and placed in a standard curing box for curing (20 °C ± 0.2, relative humidity 90%).

### 2.2. Characterization Methods

#### 2.2.1. Setting Time Test

The setting time of each fresh paste was determined using a Vicat apparatus, in accordance with the international standard ISO 9597:2008 [26].

#### 2.2.2. Flowability Test

The flowability of each fresh paste was evaluated in accordance with the Chinese standard GB/T 8077-2012 [27]. The maximum diameters of the paste in two perpendicular directions were measured with a ruler, and the average value was taken as the flowability.

#### 2.2.3. Rheology Test

**Figure 3:**
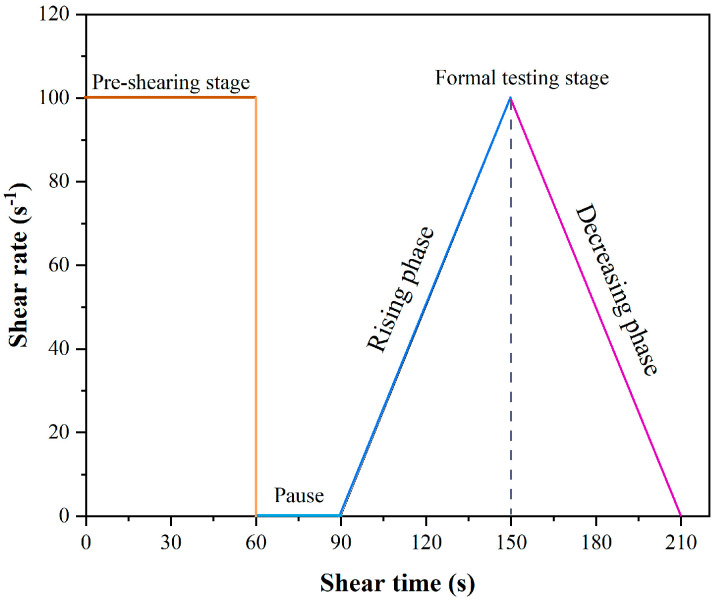
Rheological test procedure.

After 5 min of reaction, the initial and cyclic shear rheological properties of the alkali-activated paste were evaluated using a Brookfield RST 3000 rheometer (AMATEK, Inc., Berwyn, PA, USA). As illustrated in Figure 3, the initial rheological test procedure consisted of three stages: pre-shear, rest, and formal testing. In the pre-shear stage, the fresh paste was subjected to a constant shear rate of 100 s^−1^ for 60 s, followed by a 30 s rest period to reach a stable state. During the formal testing stage, the shear rate was increased linearly from 0 s^−1^ to 100 s^−1^ within 60 s and then decreased to 0 s^−1^ over the next 60 s.

#### 2.2.4. Compressive Strength Test

Compressive strength testing of samples of different ages was conducted in accordance with the international standard ISO 679:2009 [28]. Mortar specimens measuring 40 × 40 × 40 mm^3^ were tested at a loading rate of 2.4 kN/s. At least three specimens were tested for each group, and the arithmetic average for each group was calculated.

#### 2.2.5. X-Ray Diffraction (XRD) Analysis

XRD analysis of the cement paste samples was conducted using a Bruker D8 Advance instrument (Karlsruhe, Germany) with a working voltage of 40 kV, current of 40 mA, scanning range of 5–65°, and scanning speed of 2°/min.

#### 2.2.6. Fourier-Transform Infrared Spectroscopy (FTIR)

FTIR analysis was carried out using a Nicolet IS50 device (Thermo Fisher, Waltham, MA, USA). Approximately 0.05 g of a sample was placed directly on an attenuated total reflectance crystal (ATR) for FTIR measurement. Spectra were recorded in the range of 4000–500 cm^−1^, with 64 scans recorded each time, and the scan resolution was set to 4 cm^−1^. Before obtaining the spectra of each sample, a background spectrum was obtained from a clean ATR.

#### 2.2.7. Thermogravimetric Analysis (TGA)

The mass loss of the cement paste was determined using a TGA 4000 thermal gravimetric analyzer (PerkinElmer, Shelton, CT, USA). Approximately 10.0 mg of the geopolymer powder sample was taken and heated from 35 °C to 1000 °C at a heating rate of 10 °C/min.

#### 2.2.8. Scanning Electron Microscopy (SEM)

Samples were prepared for SEM as follows. Representative fragments (approximately 3–5 mm) were taken from the uncarbonized core areas of specimens that had been cured for 28 days. The fragments were immersed for 48 h in anhydrous ethanol with a purity greater than 99.5% to terminate the hydration process and replace the free water. The samples were then dried in a vacuum oven at 40 °C for 24 h until they reached a constant weight. Before observation, the dried samples were fixed to an aluminum carrier sheet using conductive tape and coated with a thin layer of gold for approximately 90 s to enhance conductivity and image quality. Morphological observation of the samples was conducted using a Hitachi TM3030 tabletop microscope (Hitachi High-Technologies Corporation, Tokyo, Japan).

## 3. Results and Analysis

### 3.1. Setting Time

**Figure 4:**
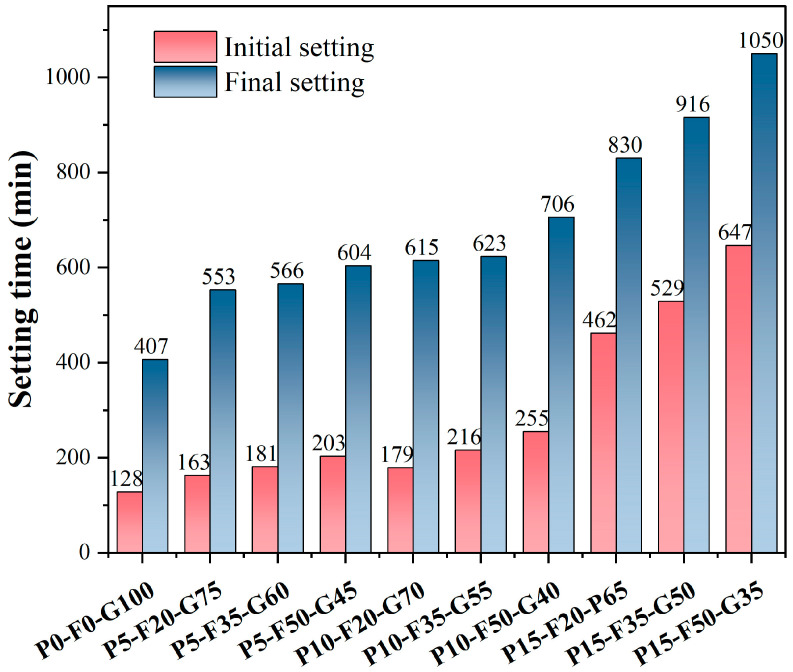
Setting time of SLPC paste.

Research on binders has shown that the setting time is a key indicator of reactivity and workability [29]. Figure 4 shows the effect of the precursor composition on the setting time of seawater-based low-alkalinity activated PG cement (SLPC) pastes. The setting time of low-alkalinity activated slurries is negatively correlated with the GGBS content, and increasing the amounts of PG and FA significantly extends the setting time. This phenomenon is consistent with the development of compressive strength. GGBS, as a highly reactive component, promotes the formation of C-A-S-H gel by releasing Ca^2+^, Si^4+^, and Al ions, thus accelerating the hardening process of the paste [30]. In contrast, the acidic characteristics of PG (pH = 2.4) have a negative impact on the setting time of the system, reducing the effective alkalinity of the alkali-activation environment. This inhibits the breaking rate of Si-O and Al-O bonds in GGBS and FA, thus delaying the reaction process [31].

Further analysis reveals that when the PG content increases to 15%, the initial setting time experiences a sharp rise (reaching up to 647 min). This is attributable, on one hand, to acid-base neutralization, and on the other hand, to the preferential nucleation and precipitation of products such as gypsum recrystallization or AFt, which consume free Si^4+^ and Al ions. These products form a physical shielding layer on the surface of GGBS and FA particles, further hindering the subsequent hydration reactions [32]. Additionally, the lower reactivity of FA results in a “dilution effect” that further delays the formation of the geopolymer network structure. Therefore, the incorporation of PG and FA not only affects the setting time of the SLPC paste but also significantly influences its early strength development. By properly adjusting the proportions of PG and FA, precise control over the construction time can be achieved. However, it is crucial to balance the relationship between setting time and strength to ensure the final material performance meets the design requirements.

### 3.2. Flowability

**Figure 5:**
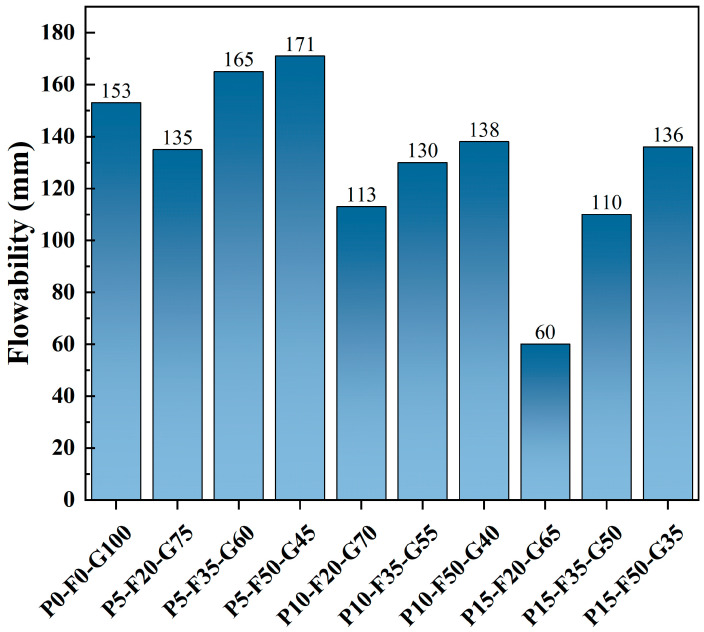
Flowability of SLPC paste.

Figure 5 shows the variation in flowability of SLPC slurries with different mix ratios. The flow performance of paste is highly sensitive to the composition of the precursor materials, particularly the incorporation of FA and PG. The addition of FA significantly improves the paste flowability. As the FA content increases, the flow diameter of the paste exhibits an upward trend. This is attributable mainly to the “ball bearing effect” of the spherical FA particles. The spherical geometry effectively reduces the internal friction between particles and acts as a lubricant during the paste flow, significantly reducing yield stress and plastic viscosity, making the paste spread more easily [33].

In contrast, the addition of PG has a noticeable negative effect on flowability. As the PG content increases, the flowability of the paste gradually decreases, because of the plate-like shape of PG particles. Unlike the spherical particles of FA, the plate-like morphology of PG significantly increases the mechanical interlocking and frictional resistance between particles, hindering the free flow of the paste [34]. Furthermore, PG typically has a larger specific surface area and higher water absorption than FA. At a given solid-to-liquid ratio, increasing the PG content leads to more free water being adsorbed onto the PG particle surfaces or into their interlayer structure, thus reducing the amount of free water available for lubrication between the particles and resulting in increased paste viscosity and decreased flowability [35,36].

GGBS particles are irregularly angular in shape, and their contribution to flowability lies between that of FA and PG [37]. When the PG content is relatively high, although the addition of FA can partially alleviate the loss of flowability, the rigid network structure formed by PG plate-like crystals tends to dominate, causing a paste with a high PG content (such as the P15-F50-G35 mix) to exhibit limited flowability despite containing a large amount of FA.

In conclusion, to achieve optimal workability that meets pumping or casting requirements, a balance must be struck between the lubricating effect of FA and the thickening effect of PG. In this study, the P5-F35-G75 mix ratio exhibited not only excellent mechanical performance but also good flowability, because of its low PG content.

### 3.3. Rheology

**Figure 6:**
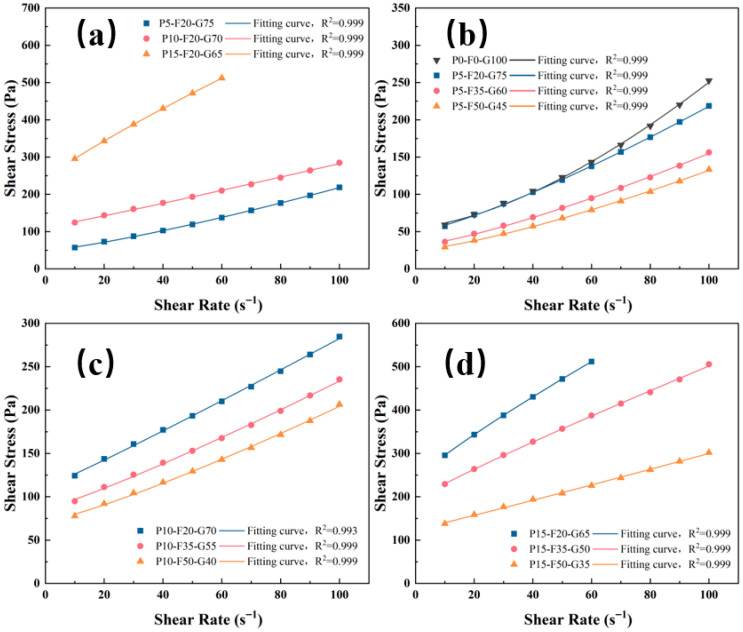
Rheological model and fitting of SLPC Paste: (**a**) Effect of PG; (**b**–**d**) Effect of FA at 5%, 10%, and 15% PG.

Figure 6 shows the shear stress–shear rate curve for the descending segment of the pastes. The Herschel–Bulkley model, shown as Equation (3), can effectively model non-Newtonian fluids with shear thickening or shear thinning characteristics:.

(3) $$\tau =\tau_{0}+\kappa \gamma^{\eta }\;\;$$

where *τ*_0_ is the yield stress and *k* is the plastic viscosity. A detailed discussion of the impact of ternary activators on rheological properties is of great significance in understanding the complex interactions between seawater components and activator anions.

As Table 5 shows, the coefficients of determination (R^2^) of all fitted rheological parameters were greater than 0.99, indicating that the H-B model accurately describes the flow characteristics of the seawater-based PG-FA-GGBS ternary system [38]. Figure 6a shows that the addition of PG significantly degrades the rheological parameters, which is consistent with the flowability results. When the FA content remained constant, as the PG content increased from 5% to 15%, the yield stress of the paste increased from 49.19 to 238.01 Pa, while the plastic viscosity increased from 0.52 to 7.77 Pa·s. The plate-like crystal structure of PG particles increases the internal friction of the paste and enhances mechanical interlocking between particles, thus requiring higher shear stress for the paste to flow again. More critically, the rapid dissolution of PG in the seawater environment releases high concentrations of Ca^2+^ and SO_4_^2−^ [39], promoting the formation of a large amount of early AFt. The interwoven needle-like AFt crystals combine with a large amount of free water, further restricting the free flow of the paste [40].

In contrast to PG, the addition of FA effectively improves the plasticity of the system, acting as a rheological modifier. Figure 6b,c show that for groups with the same PG content, increasing the FA content significantly reduces shear stress and plastic viscosity. At 15% PG, the use of 50% FA reduced the viscosity by 81.6%. FA also has relatively low reactivity in the initial stage (compared to GGBS), which means that less free water is consumed quickly, leaving more water available to lubricate the paste [41]. Notably, the 5-20-75 mix ratio is slightly lower than the pure GGBS control group. This indicates that at low dosages (5%), the adverse morphological effects of PG can be compensated by the lubricating effect of FA. Therefore, in practical engineering applications, the operability can be adjusted by controlling the amounts of PG and FA.

### 3.4. Phase Analysis

#### 3.4.1. FTIR Analysis

**Figure 7:**
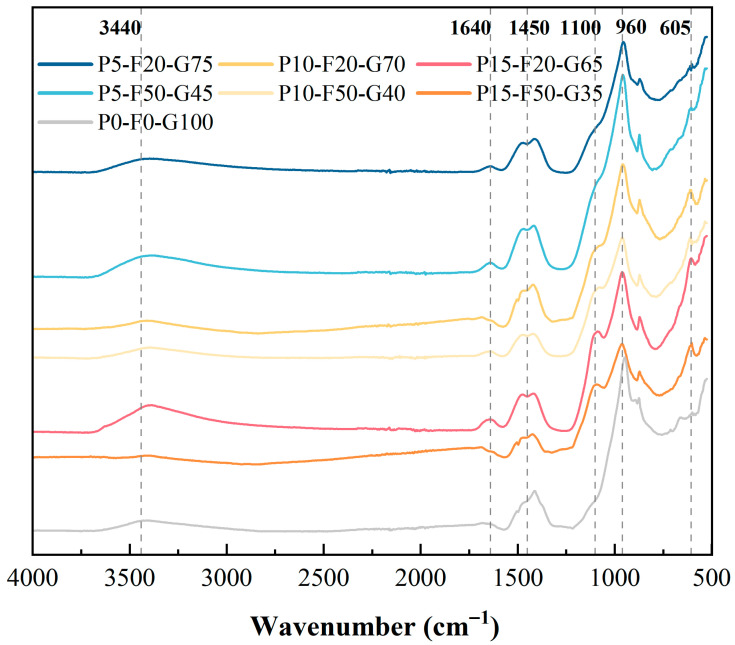
FTIR spectra of SLPC paste.

Figure 7 shows the FTIR spectra of the SLPC paste cured for 28 days, with absorption bands revealing the formation of functional groups and hydration products in the gel matrix. In all samples, a broad absorption band was observed around 3440 cm^−1^, and a weak peak was detected around 1640 cm^−1^. These are attributable to the stretching and bending vibrations of the O-H group. The presence of these bands indicates the existence of chemically bound water in hydration products such as C-A-S-H gel and AFt, as well as free water in the pore solution [10].

An evident absorption peak appeared in the 1420–1460 cm^−1^ range, corresponding to the asymmetric stretching vibration of the O-C-O bond in the carbonate group (CO_3_^2−^). This is mainly due to the use of Na_2_CO_3_ in the composite activator and the inevitable carbonation of the matrix during curing and sample preparation [42]. The formation of main products is primarily concentrated in the 900–1200 cm^−1^ spectral region. For the control group (P0-F0-G100), a sharp peak appeared around 960 cm^−1^, which is a typical characteristic of C-(A)-S-H gel formed during GGBS hydration [43]. With the incorporation of PG and FA, this main peak broadened, and its intensity changed. The broadening of the peak indicates that, because of the interactions between various precursors, the gel structure became more disordered, and different phases coexisted [44]. Also, phosphorus released from PG could be incorporated into the aluminosilicate network structure, similar to silicon and aluminium. Among them, Al and P exhibited high exchangeability and readily formed P–O–Al bonds [45].

It is noteworthy that in the samples containing PG (such as P5-F20-G75 and P15-F20-G65), a distinct shoulder peak or band broadening was observed at 1100–1200 cm^−1^. This is primarily attributable to the stretching vibration of the SO_4_^2−^ group [32]. The detection of sulfate confirms the dissolution of PG, where sulfate ions participate in reactions to form AFt. The presence of these sulfate phases acts as a framework, densifying the matrix and contributing to the development of strength [46]. Furthermore, in the low-wavenumber region, especially in the spectra of PG-containing mixes (such as P15-F20-G65 and P5-F20-G75), a sharp absorption band appears around 605 cm^−1^. This band corresponds to the O-S-O bending vibration of the sulfate tetrahedron (SO_4_^2−^). In the control group (P0-F0-G100), the bands in the 500–800 cm^−1^ range are relatively broad and shallow, mainly corresponding to the bending vibration of the Al–O bond in the aluminosilicate framework. However, the sharp peak at 605 cm^−1^ in the ternary system is a diagnostic fingerprint characteristic of the crystalline sulfate phase. This provides dual evidence: it indicates the presence of residual gypsum from PG and, more importantly, confirms the formation of AFt, as AFt exhibits a characteristic sulfate bending mode in this frequency range [47]. This structural observation is consistent with the mechanical strength results, confirming that the sulfate groups provided by PG are effectively incorporated into the hydration products, forming an AFt-enhanced matrix.

#### 3.4.2. XRD Analysis

**Figure 8:**
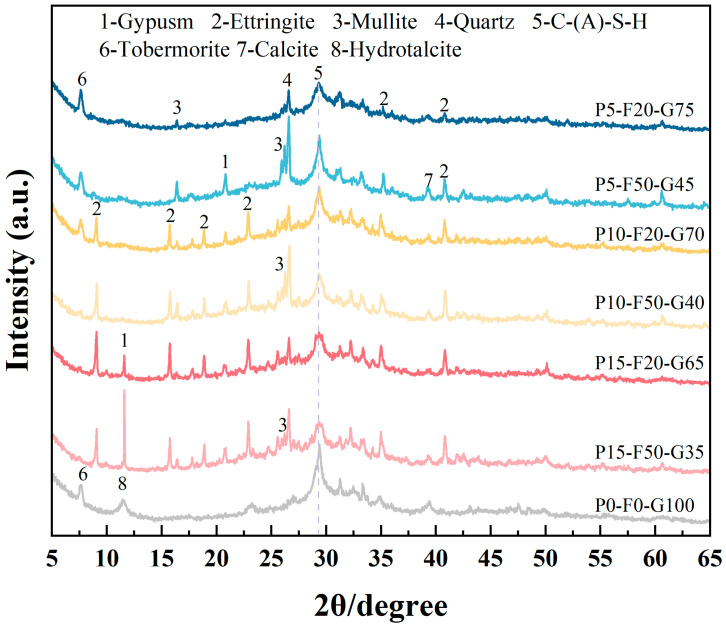
XRD spectra of SLPC paste.

Figure 8 shows the X-ray diffraction (XRD) patterns of slurries with different mix ratios at 28 days. All samples exhibit a strong peak around 29° (2θ), indicating the formation of a significant amount of amorphous C-(A)-S-H gel. This amorphous gel constitutes the main binding phase in the system and plays an important role in the macroscopic mechanical properties [48]. For the control group (P0-F0-G100), the intensity of this peak is relatively high, consistent with this group having the highest compressive strength. In the P5-F20-G75 group, characteristic diffraction peaks of AFt were detected at 2θ = 35° and 41°, confirming that the SO_4_^2−^ provided by 5% PG actively participated in the hydration reaction, reacting with dissolved Al^3+^ and Ca^2+^ in GGBS to form crystalline AFt [49]. Notably, the characteristic peak of gypsum in this group is nearly invisible, indicating that the added PG has been almost completely consumed [50]. These AFt crystals, acting as micro-reinforcements, intertwine with the gel matrix, compensating for the strength loss caused by the reduction in GGBS content [51].

It is worth noting that no crystalline phases of soluble Ca_3_(PO_4_)_2_ or CaF_2_ were detected in the 28-day samples. As shown in the FTIR analysis in the previous section, the chemical bonding of the gel network can effectively absorb harmful substances such as phosphorus. Previous studies have shown that the C-(A)-S-H gel network has strong ion exchange capabilities and can be substituted by similar cations or anions, thus achieving chemical bonding [52]. In addition to chemical bonding, harmful ions in PG can also be immobilized physically. For example, lattice distortion of the AFt crystal structure can be caused by interstitial doping, forming a solid solution [53,54]. Although PG introduces more impurities, the large amount of AFt formed enables the self-gelling and solidification of harmful ions. This indicates that SLPC, as an environmentally friendly material, has great potential to replace traditional cement-based materials.

In contrast, for the P15-F50-G35 mix ratio, strong diffraction peaks corresponding to gypsum were observed, along with quartz and mullite peaks from the unreacted FA. The continued presence of the gypsum phase indicates that excess calcium phosphate failed to fully dissolve and transform, remaining in the matrix as an inert filler [55]. These unreacted phases disrupt the continuity of the gel network, leading to low strength and a loose microstructure [56]. At PG contents of 5% and 10%, the characteristic peak of AFt significantly increases as the FA content increases, suggesting that more FA provides additional free Al to the matrix. However, when the PG content exceeds 15%, the formation of AFt is hindered as FA increases, because of the further decline in system reactivity.

#### 3.4.3. TGA

**Figure 9:**
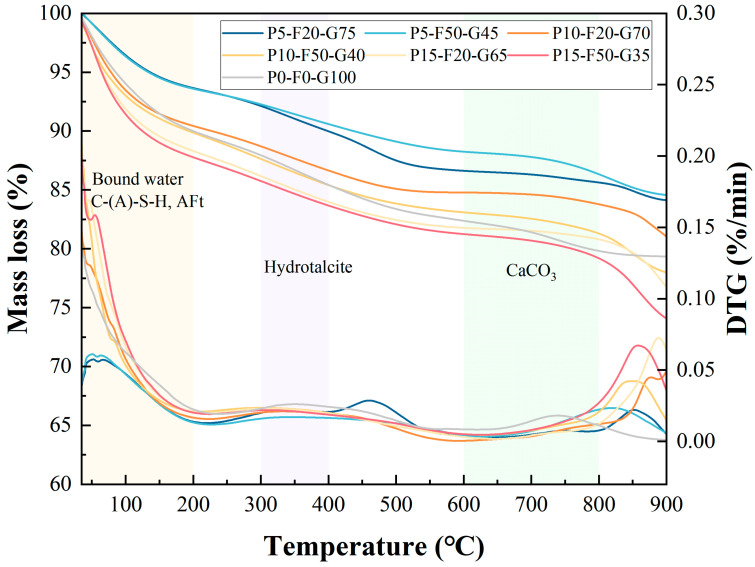
TG and DTG curves of the SLPC paste at 28 days.

Figure 9 shows the TG and DTG curves of the SLPC paste at 28 days of curing. The thermal analysis curves not only quantify the thermal stability of the reaction products but also further validate the conclusions regarding the types of hydration products from the FTIR and XRD analyses. Based on the weight loss characteristics, the thermal decomposition process of the samples can be divided into three main temperature ranges: 50–200 °C, 200–600 °C, and 600–800 °C [57,58].

The most significant weight loss occurred in the 50–200 °C range, primarily because of the dehydration of amorphous gel products (C-(A)-S-H) and crystalline hydration products such as gypsum and AFt [59]. The higher weight loss observed in the control group (P0-F0-G100) is mainly attributable to C-(A)-S-H gels, confirming that the pure GGBS system has the highest degree of hydration and gel formation. Additionally, for the PG-containing groups, the weight loss in this temperature range also includes the loss of crystalline water in AFt (90–110 °C) [59]. This corresponds with the S-O vibration observed at 1100 and 605 cm^−1^ in the FTIR analysis, confirming that the sulfate ions provided by PG effectively participated in the reaction, promoting the formation of AFt crystals, thereby filling pores and contributing to strength [46].

In the 200–600 °C range, the weight loss rate became more gradual, involving the release of more stable bound water. Considering that seawater contains a higher concentration of Mg^2+^ (as shown in Table 2) and MgO components in GGBS, the weight loss around 300–400 °C could be attributed to the removal of interlayer water from hydrotalcite and the hydroxyl oxidation [60]. This suggests that magnesium ions in seawater not only exist in physical form but also partially participate in chemical bonding [61]. In this range, the P0-F0-G100 group shows clear hydrotalcite crystallization in the XRD pattern, unlike the other groups, possibly because of interference from the gypsum phase.

In the 600–800 °C range, the weight loss mainly corresponds to the decomposition of calcite (CaCO_3_). The DTG curve shows a weight loss peak between 650 and 750 °C, confirming the presence of CaCO_3_. This is partly due to the use of Na_2_CO_3_ in the composite activator and partly due to the inevitable carbonation during sample preparation and curing [42]. It is noteworthy that the incorporation of FA typically results in a slight decrease in the total weight loss. This is because the pozzolanic reactivity of FA is lower than that of GGBS at room temperature, leading to the formation of fewer gel products, and the unreacted FA particles, acting like micro-aggregates, fill the matrix and remain thermally stable at high temperatures. The “dilution effect” produced by FA reduces the volatile components in the unit mass of the sample [62].

Additionally, although the thermal decomposition of anhydrous calcium sulfate typically occurs above 1200 °C, the characteristic peak observed around 900 °C has significant chemical implications. This weight loss is attributable to the instability and decomposition of anhydrous calcium sulfate (from unreacted PG or AFt decomposition products) [63]. In a matrix rich in SiO_2_ and Al_2_O_3_, CaSO_4_ undergoes a solid-state reaction with these oxides at high temperatures, significantly lowering the decomposition starting temperature of the sulfate. Furthermore, trace impurities remaining in PG (such as fluorides and phosphates) may act as fluxing agents, further promoting lattice disruption and the release of SO_x_ gases [64]. This suggests that the system chemically bonds the sulfur in PG at room temperature but that the chemical interactions between the silicate matrix and sulfates limit its thermal stability in extremely high-temperature environments.

#### 3.4.4. Morphology

**Figure 10:**
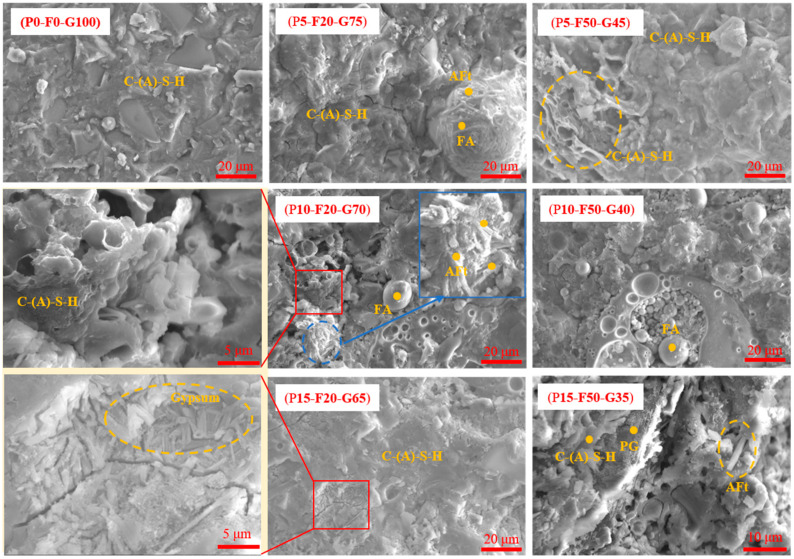
SEM Images of the SLPC paste at 28 days.

Figure 10 shows the SEM microstructures of the samples at 28 days of curing. The control group exhibited a highly dense and uniform microstructure. A large amount of C-(A)-S-H gel is densely packed, forming a continuous, cohesive matrix, with almost no noticeable pores or cracks. This indicates that under the action of the composite alkaline activator, the highly reactive GGBS particles underwent full dissolution and polycondensation reactions, producing a substantial amount of gel. This dense microstructure effectively fills the space, providing the matrix with excellent load-bearing capacity [65], which is consistent with this group having the highest compressive strength. In contrast, the P5-F20-G75 group, containing an appropriate amount of PG and FA, displays a unique “crystal–gel” structure. A large number of needle-like crystals are clearly observed, interspersed and tightly growing around FA particles. It is evident that the Al leached from FA facilitates the preferential nucleation and growth of AFt crystals [49]. Similarly, these needle-like AFt crystals act like “microfibers” embedded within the gel pores, bridging and filling the gaps and enhancing the compactness of the matrix [66]. Additionally, some FA particles, which show signs of surface erosion, are embedded in the matrix, further densifying the overall structure.

For the groups with high FA contents, such as the P5-F50-G45 mix, large numbers of gel structures with pores can be observed. This is because a reduction in GGBS content leads to a decrease in the total amount of gel formation. However, unreacted FA spheres are tightly embedded in the matrix, acting as micro-aggregates, filling the gaps in the gel network and suppressing the propagation of microcracks [67]. This explains why the P5-F50-G45 group had a compressive strength of more than 50 MPa despite the high substitution rate. However, in the high PG and FA groups (P10-F50-G40/P15-F50-G35 groups), the microstructure showed significant deterioration. SEM images reveal a loose matrix structure with obvious pores and microcracks. A large number of unreacted smooth spherical FA particles and plate-like PG crystals are loosely agglomerated, with weak interfacial bonding between the particles and the gel matrix [68].

In conclusion, the SEM analysis confirms that the growth of AFt crystals activated by an appropriate amount of PG (5%) can effectively refine the pore structure. However, excessive incorporation of PG leads to a loose structure and insufficient gel formation, which is highly consistent with the macroscopic mechanical performance and phase analysis results.

### 3.5. Compressive Strength

**Figure 11:**
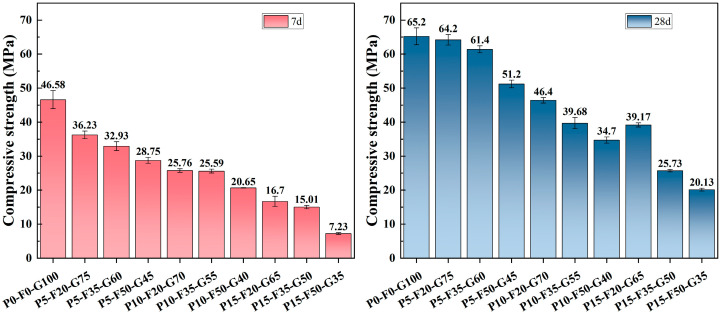
Compressive Strength of SLPC paste at 7 and 28 Days.

Figure 11 shows the development of compressive strength for SLPC at 7 and 28 days of curing. Overall, the compressive strength of all samples consistently increased with curing time because of ongoing hydration. The control group (P0-F0-G100), which consists of pure GGBS, exhibited the highest compressive strength at all curing ages. However, mixes with PG and FA incorporated had lower strengths than the pure GGBS matrix, indicating a complex interaction between these waste variants and the binder system. As the PG content increased, a significant decrease in strength was observed. This phenomenon can be attributed to the presence of impurities in the untreated PG, such as soluble phosphorus and fluoride. These impurities adhere to the surface of the GGBS particles, forming a passivating layer that hinders the dissolution of Si^4+^ and Al^3+^ ions, thereby slowing down the hydration process [39]. Additionally, the acidity (pH) of PG neutralizes the alkalinity provided by the weak alkaline activator, reducing the reaction driving force required for the decomposition of GGBS glass and FA [24,58]. Notably, for the P5-F50-G45 mix ratio, although the compressive strength was slightly lower than that of the control group and the PG group with the same PG content, the strength at 28 days exceeded 50 MPa, indicating remarkable mechanical performance. This may be due to the high salinity of seawater, which promotes the further dissolution of GGBS glass and FA. Ashraf et al. [69] noted that the high salinity characteristics of seawater facilitate the dissolution of high-alumina precursors. A relatively low dosage (5%) of PG can provide sufficient Ca^2+^ [31], which is beneficial for the formation of the main product, C-(A)-S-H gel.

For a given PG content, the compressive strength decreases within a relatively small range as the FA content increases. This phenomenon is primarily due to the lower reaction activity of FA at room temperature compared to GGBS. FA is a pozzolanic material with a slower reaction rate. Increasing the proportion of FA to GGBS leads to a “dilution effect” of calcium content, resulting in a more porous microstructure and fewer hydration products during the early and middle stages of curing [62]. Therefore, replacing reactive GGBS with FA to some extent causes changes in strength. Although high doses of PG and FA each produce negative effects, the system demonstrates significant potential under specific mix designs. For example, the sample 5-35-60 reached a compressive strength of 61.4 MPa at 28 days, which is comparable to that of the control group at 65.2 MPa. This indicates the existence of a synergistic effect in seawater-based low-alkalinity systems. Seawater not only serves as mixing water but also acts as an ion supplement, providing alkali metal ions (Na^+^, Mg^2+^, and Ca^2+^) and anions (Cl^−^, SO_4_^2−^, and HCO_3_^−^). Several studies have shown that cations in seawater can undergo exchange reactions with precursors, especially GGBS, thereby promoting the hydration reaction of the system [15,60]. These ions can guide and participate in the further formation of byproducts such as Friedel’s salts and AFt, filling capillary pores [70]. Additionally, the composite activator creates a specific alkaline chemical environment in which sulfate and 5% PG together promote the formation of AFt. This AFt acts as a reinforcing framework, connecting with C-(A)-S-H gel and making the matrix more compact [66]. However, this balance is delicate. As seen in the high PG group (15%), it leads to insufficient reactivity and a sharp decline in mechanical performance.

## 4. Conclusions

This article proposes a novel preparation method for seawater-based low-alkalinity PG cement that takes advantage of the synergistic reactivity of a GGBS-FA-PG multi-waste system in a highly ionic seawater environment. This method addresses the issue of PG stockpiling while overcoming the problems of excessive alkalinity and cost associated with traditional high-alkali activation. The following key conclusions were drawn:

1. This preparation method effectively regulates the rheological behavior and hardening performance of the multi-waste system. The results show that low-alkalinity seawater mixing significantly improves the flowability of the paste. Specifically, when the PG content is 5% and the FA content is 35%, the material demonstrates excellent mechanical performance, with a 28-day compressive strength comparable to that of the pure GGBS group, and the setting time and workability meet engineering requirements.
2. The seawater environment alters the hydration kinetics of SLPC. In the early stages of hydration, the ions in seawater act as a reactive medium, accelerating the dissolution of precursors and directly participating in the rapid nucleation of products such as AFt and hydrotalcite. At the same time, the low-alkalinity environment prevents rapid precipitation and induces the formation of aluminum-rich C-(A)-S-H gel.
3. SEM confirmed the formation of a unique “crystal–gel” structure in the later stages of hydration. Needle-like AFt crystals are tightly intertwined with the amorphous C-(A)-S-H gel matrix. This dual action of physical filling and chemical bonding significantly refines the pore structure, reducing the proportion of harmful pores and imparting excellent macroscopic mechanical strength to the matrix.
4. The enhancement of SLPC compressive strength is primarily attributable to the synergy between the seawater ion enhancement effect and the low-alkalinity complementary mechanism. The active ions in seawater effectively compensate for the insufficient activation ability under low-alkalinity conditions, stabilizing the main hydration products. Additionally, by solidifying the harmful impurities in PG, seawater transforms them into mineral phases that are beneficial to strength.

## Figures and Tables

**Table 1:** Chemical compositions of the raw materials.

Oxide	CaO (%)	SiO_2_ (%)	Al_2_O_3_ (%)	MgO (%)	Fe_2_O_3_ (%)	K_2_O (%)	Na_2_O (%)	SO_3_ (%)	P_2_O_5_ (%)	Others (%)
GGBS	39.26	28.09	15.96	8.70	0.27	0.33	3.66	1.40	/	2.33
FA	3.36	54.36	33.46	1.1	3.24	2.03	0.446	0.318	0.152	1.534
PG	37.56	6.75	0.87	0.24	0.51	0.53	2.78	47.53	1.94	1.29

**Table 2:** The pH of seawater mixed with Na_2_SO_4_, Na_2_CO_3_, and Na_2_SiO_3_ powders.

Compound	pH
Na_2_SO_4_	7.85
Na_2_CO_3_	10.94
Na_2_SiO_3_	13.65

**Table 3:** Mixture proportions of precursors (g) and chemical composition of the ternary activator (g).

Mixture	PG	FA	GGBS	N_2_O	SO_3_	CO_2_	SiO_2_	Water
P0-F0-G100	/	/	100	4	1.55	0.57	2.14	40
P5-F20-G75	5	20	75	4	1.55	0.57	2.14	40
P5-F35-G60	5	35	60	4	1.55	0.57	2.14	40
P5-F50-G45	5	50	45	4	1.55	0.57	2.14	40
P10-F20-G70	10	20	70	4	1.55	0.57	2.14	40
P10-F35-G55	10	35	55	4	1.55	0.57	2.14	40
P10-F50-G40	10	50	40	4	1.55	0.57	2.14	40
P15-F20-G65	15	20	65	4	1.55	0.57	2.14	40
P15-F35-G50	15	35	50	4	1.55	0.57	2.14	40
P15-F50-G35	15	50	35	4	1.55	0.57	2.14	40

Notes: P = PG, F = FA, G = GGBS.

**Table 4:** Chemical composition of artificial seawater, from ASTM D1141 (2021) [25].

Compound	NaCl	MgCl_2_	Na_2_SO_4_	CaCl_2_	KCl	NaHCO_3_	KBr	H_3_BO_3_	SrCl_2_	NaF
Concentration (g/L)	24.53	5.20	4.09	1.16	0.695	0.201	0.101	0.027	0.025	0.003

**Table 5:** Rheological parameters of SLPC paste.

Ground	τ_0_ (Pa)	k (Pa·s)	R^2^
P0-F0-G100	56.18	0.15	0.999
P5-F20-G75	49.19	0.52	0.999
P5-F35-G60	31.15	0.31	0.999
P5-F50-G45	25.21	0.23	0.999
P10-F20-G70	111.42	1.28	0.993
P10-F35-G55	86.28	0.77	0.999
P10-F50-G40	71.65	0.52	0.999
P15-F20-G65	238.01	7.77	0.999
P15-F35-G50	192.20	4.61	0.999
P15-F50-G35	124.53	1.43	0.998

## Data Availability

The original contributions presented in this study are included in the article. Further inquiries can be directed to the corresponding author.
